# Mechanisms underpinning sympathetic nervous activity and its modulation using transcutaneous vagus nerve stimulation

**DOI:** 10.1113/EP086433

**Published:** 2017-12-03

**Authors:** Susan A. Deuchars, Varinder K. Lall, Jennifer Clancy, Mohd Mahadi, Aaron Murray, Lucy Peers, Jim Deuchars

**Affiliations:** ^1^ School of Biomedical Science, Faculty of Biological Science University of Leeds Leeds UK; ^2^ Faculty of Pharmacy Universiti Kebangsaan Malaysia Kuala Lumpur Malaysia

**Keywords:** sympathetic nervous system, neuromodulation, spinal cord, gap junction, vagus nerve stimulation

## Abstract

**New Findings:**

- **What is the topic of this review?**
  This review briefly considers what modulates sympathetic nerve activity and how it may change as we age or in pathological conditions. It then focuses on transcutaneous vagus nerve stimulation, a method of neuromodulation in autonomic cardiovascular control.
- **What advances does it highlight?**
  The review considers the pathways involved in eliciting the changes in autonomic balance seen with transcutaneous vagus nerve stimulation in relationship to other neuromodulatory techniques.

The autonomic nervous system, consisting of the sympathetic and parasympathetic branches, is a major contributor to the maintenance of cardiovascular variables within homeostatic limits. As we age or in certain pathological conditions, the balance between the two branches changes such that sympathetic activity is more dominant, and this change in dominance is negatively correlated with prognosis in conditions such as heart failure. We have shown that non‐invasive stimulation of the tragus of the ear increases parasympathetic activity and reduces sympathetic activity and that the extent of this effect is correlated with the baseline cardiovascular parameters of different subjects. The effects could be attributable to activation of the afferent branch of the vagus and, potentially, other sensory nerves in that region. This indicates that tragus stimulation may be a viable treatment in disorders where autonomic activity to the heart is compromised.

## Introduction

The autonomic nervous system is pivotal in homeostatic control of many end organs. For organs that receive dual innervation from the sympathetic and parasympathetic branches of the autonomic nervous system, such as the heart, eyes, bladder, bowel and sex organs, it provides a way in which fine control of activity can be managed by altering the balance of activity in the two branches that mainly act reciprocally. The extent to which each branch contributes to the overall level of activity varies throughout life, with differences observed during healthy ageing but also in specific conditions, such as heart failure, where sympathetic activity predominates and may contribute to the detrimental effects on cardiovascular function. Our aim is to understand what contributes to resting sympathetic activity and how we may restore autonomic balance in these situations where sympathetic activity is abnormally raised or parasympathetic activity is lowered, potentially enabling simple novel interventions to be used as alternative or adjunct therapies in such conditions.

## Sympathetic activity at rest

The sympathetic nervous outflow is regulated by complex CNS circuitry that receives afferent information and culminates in outflow from the CNS through sympathetic preganglionic neurones located in the thoracolumbar regions of the spinal cord. The axons of these neurones exit the spinal cord to innervate postganglionic neurones that in turn innervate their target organs (Deuchars & Lall, 2015). The activity of sympathetic preganglionic neurones dictates the level of excitability in the outflow to different end organs, which needs to be kept within homeostatic limits.

**Figure 1:**
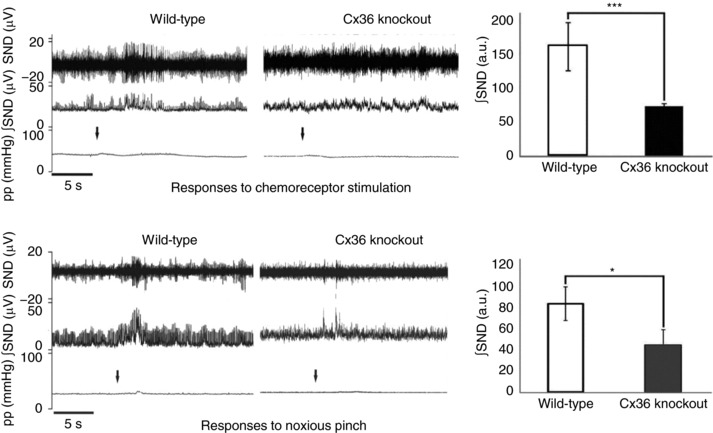
Sympathetic and heart rate responses to chemoreceptor or noxious stimulation are blunted in the connexin 36 knockout (KO) mouse Each panel shows the raw data, obtained using the working heart–brainstem preparation, with sympathetic nerve discharge (SND), integrated sympathetic nerve activity (∫SND) and perfusion pressure (pp) and the mean data for integrated sympathetic nerve discharge. Sympathoexcitation upon chemoreceptor stimulation or noxious pinch (arrow denotes point of stimulation) is significantly reduced in Cx36 KO mice compared with wild‐type. Adapted from Lall *et al*. (2017), with permission.

There are a number of influences converging onto sympathetic preganglionic activity. Sensory information from specialized receptors causes reflex changes (involving either spinal or supraspinal circuits) in sympathetic activity that act to enable the body to respond appropriately to the original stimulus, whether this is stretching of baroreceptors because of a change in blood pressure or a noxious stimulus that requires an autonomic component to the response. Other descending pathways from a number of brain regions contribute to the overall activity, and the extent of this influence may alter in situations of mental stress (Callister *et al*. 1992), pathologies such as heart failure (discussed below) or as a circadian change (Grassi *et al*. 2010). Many of these pathways involve interneurones, some of which are in close vicinity to the sympathetic outflows (Deuchars & Lall, 2015). These sympathetic preganglionic neurones are also electrically coupled through gap junctions composed of connexin 36 subunits that enable appropriately synchronized responses to specific perturbations (Lall *et al*. 2017). Knockout of connexin subunits increases variance in blood pressure and heart rate in conscious mice, especially when a running wheel is introduced into their cage. Furthermore, in anaesthetized animals or in the anaesthetic‐free working heart–brainstem preparation, the sympathetic or cardiovascular responses to sensory stimulation, such as activation of peripheral chemoreceptors or noxious pinch, are significantly reduced (Fig. 1). It is, therefore, clear that sympathetic activity is precisely controlled through both intrinsic and extrinsic mechanisms, and activation of these different inputs may enable modulation of this activity when required.

## The character of cardiovascular autonomic activity is related to age

**Figure 2:**
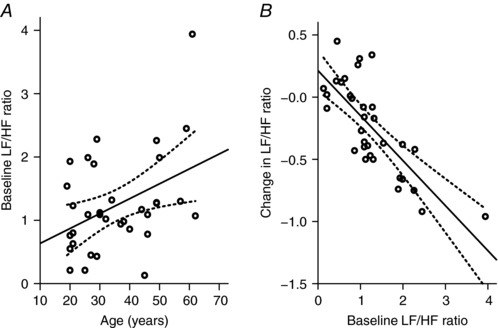
Relationship between low‐frequency to high‐frequency (LF/HF) ratio and effects of transcutaneous vagus nerve stimulation *A*, plotting the baseline LF/HF ratio against age shows a significant relationship between these parameters (*R* ^2^ = 0.19; *P* = 0.013). *B*, there is a relationship between baseline LF/HF ratio and change in LF/HF ratio during transcutaneous vagus nerve stimulation, indicating that higher LF/HF ratios predict a greater decrease in LF/HF during transcutaneous vagus nerve stimulation (*R* ^2^ = 0.58; *P *< 0.0005). Adapted with permission from Clancy *et al*. (2014).

Cardiovascular health is dictated by changes in autonomic activity that lead to altered blood pressure, cardiac function and responses to exercise, for example. The contributions of the different branches of the autonomic nervous system to cardiovascular autonomic function are often assessed non‐invasively in humans using heart rate or blood pressure variability analysis. Heart rate varies from beat to beat, and these variations reflect parasympathetic and sympathetic inputs to the sinoatrial node. The high‐frequency component of heart rate variability is considered to be related to rapid variations in heart rate associated with vagally mediated respiratory sinus arrhythmia (Malliani, 2005). In conscious dogs, this component is abolished by parasympathetic blockade using glycopyrrolate (Akselrod *et al*. 1981). Research suggests that as people age, the contribution of the parasympathetic activity to the heart declines, whereas sympathetic tone increases. Using heart rate variability and changes in respiratory sinus arrhythmia as measures of vagal tone (De Meersman & Stein, 2007; Abhishekh *et al*. 2013), there is a decline in parasympathetic control of the heart. Concomitantly, with ageing, there is an increase in sympathetic activity, measured as higher plasma noradrenaline concentrations (Esler *et al*. 1995) or increased muscle sympathetic activity (Ebert *et al*. 1992; Ng *et al*. 1993). Our research has also shown this positive correlation between age and baseline heart rate variability (Fig. 2). This change in autonomic balance is associated with an increased likelihood of developing cardiovascular disease (Umetani *et al*. 1998; Abhishekh *et al*. 2013), and thus, restoration of this balance in ageing has merited consideration as a therapeutic target.

## In cardiovascular disease, such as heart failure, autonomic function is also affected

Many people in the UK today are living with a diagnosis of heart failure that can be described simply as a reduction in pumping capacity of the heart but in fact is a complex syndrome arising from many different causes, such as a heart attack, high blood pressure or cardiomyopathy. It is well established that in heart failure, there is a chronic elevation in sympathetic activity, measured as increased plasma noradrenaline concentrations (Cohn *et al*. 1984; Kaye *et al*. 1995) or muscle sympathetic activity (Macefield *et al*. 1999; Barretto *et al*. 2009); both measurements positively correlated with increased mortality. There is also vagal withdrawal (Eckberg *et al*. 1971; Imai *et al*. 1994), suggesting impaired parasympathetic function. Overall, therefore, heart rate variability is impaired in heart failure patients (Nolan *et al*. 1998), with such a close correlation with mortality rate that it may be considered a predictor of prognosis after myocardial infarction (La Rovere *et al*. 1998).

## Non‐invasive stimulation of the tragus changes autonomic balance

Neuromodulation is fast becoming a technique considered as a therapeutic avenue in conditions as diverse as obesity, urinary function and chronic pain. Similar methods, such as vagus nerve stimulation, spinal cord stimulation, renal denervation or baroreceptor activation, have been studied for treatments of heart failure but with mixed outcomes at clinical trial (Schwartz *et al*. 2015). In a bid to identify non‐invasive mechanisms to maintain or restore autonomic balance to healthy levels, we investigated the effects of stimulating nerves that run in the outer parts of the ear, based on previous studies that suggested that electroacupuncture of this region could reduce the dependence of patients with coronary artery disease on their vasodilator medication (Zamotrinsky *et al*. 1997, 2001) and increase their exercise tolerance. We found that stimulation of the tragus part of the ear, using specific stimulation parameters established in the laboratory (200 μs pulses at 30 Hz 10–50 mA, which was slightly below the level at which subjects could feel the stimulus) significantly decreased the low‐frequency to high‐frequency ratio (LF/HF) of the heart rate variability, indicative of an increase in parasympathetic activity. Furthermore, by measuring muscle sympathetic nerve activity directly, using microneurography and specific tests to identify individual units as being of sympathetic origin, stimulation of the tragus significantly reduced the incidence of firing of single sympathetic fibres (Clancy *et al*. 2014). These results suggested that autonomic balance was being altered by this intervention to increase the vagal contribution. The extent of effectiveness of this simple treatment was clearly seen by the significant correlation between the magnitudes of the effects compared with the resting LF/HF ratio of the subjects (Fig. 2). This suggests that in conditions with higher resting sympathetic contribution to heart rate variability, such as ageing or heart failure, it is more likely that this treatment will restore balance towards a vagal influence. Indeed, our most recent cohort of subjects, taken from both ageing populations and those with heart failure, are consistent with these observations.

## What are the pathways underlying these effects on heart rate variability?

If we are to understand fully whether such an intervention can be useful in the treatment of conditions such as heart failure, it is crucial to elucidate the pathways being activated by this transcutaneous vagus nerve stimulation. This is particularly pertinent when one considers the recent clinical trials of whole‐nerve vagus nerve stimulation in the treatment of conditions such as heart failure, where the results were less promising than hoped (Byku & Mann, 2016). This prompted some extensive reflection on the reasons behind the disappointing results. One major difference between the trials concerns the stimulation parameters (both amplitude of current and time of stimulation), a huge consideration when one considers that the nerve being activated is formed of small‐diameter unmyelinated C fibres along with myelinated A and B fibres (Byku & Mann, 2016), all of which have different activation thresholds. Thus, for each trial, a different assortment of fibres may be stimulated. Furthermore, the vagus nerve contains both afferent and efferent fibres, and some studies were designed preferentially to activate the efferent side, yet studies in cats report that in fact afferent vagal stimulation significantly inhibits sympathetic activity (Schwartz *et al*. 1973), and this may therefore be a more relevant therapeutic avenue to ameliorate the increase in cardiac sympathetic outflow to the heart. Perhaps a greater issue is the recent report that the cervical vagus in humans may also contain sympathetic fibres (Verlinden *et al*. 2016), so stimulating this nerve at the neck may concomitantly activate sympathetic and parasympathetic efferent fibres.

**Figure 3:**
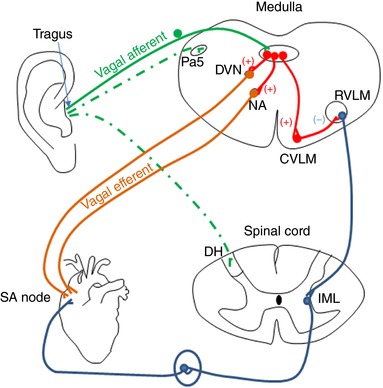
Potential pathways involved in cardiovascular responses elicited by stimulation of the tragus The pathways in green represent the potential afferent inputs to the CNS, with the dotted lines being those under investigation. Red pathways are interneurones involved in the autonomic modulation, while vagal efferent pathways are in orange and the sympathetic components of the pathways are in blue. Abbreviations: Pa5, paratrigeminal nucleus; DVN, dorsal vagal motor nucleus; NA, nucleus ambiguus; RVLM, rostral ventrolateral medulla; CVLM, caudal ventrolateral medulla; DH, dorsal horn; IML, intermediolateral cell column; SA Node, sinoatrial node.

Choosing a neuromodulatory regime that preferentially activates afferent fibres may therefore be more effective. The site at which we stimulate the ear is one that has since been tested for treatment of atrial fibrillation (Stavrakis *et al*. 2015) as well as conditions that are not cardiovascular related, such as chronic pain, tinnitus and epilepsy (Clancy *et al*. 2013; Murray *et al*. 2016). The tragus is innervated by a purely sensory branch of the vagus nerve, known as the auricular branch of the vagus, which could explain the significant sympatho‐inhibitory effects observed with stimulating this region, considering the findings of Schwartz *et al*. (1973) considered above. However, this region of the ear is also innervated by the great auricular nerve and the auriculotemporal nerve (Peuker & Filler, 2002), so it is not necessarily solely vagus nerve stimulation that is eliciting these effects. Functional MRI studies have shown in the brain that transcutaneous vagus nerve stimulation (at tragus, 250 μs pulse width, 4–8 mA) activates regions of the brain associated with the vagal afferent input synaptic pathway, such as the locus coeruleus of the brainstem, the thalamus, the prefrontal cortex, the postcentral gyrus, the posterior cingulate gyrus and the insula cortex (Dietrich *et al*. 2008). These regions were mainly ipsilaterally activated, and their responses differed from that of Kraus *et al*. (2007), probably owing to differences in stimulation parameters (20 μs pulse width, 8 Hz). Moreover, no focus has been placed on spinal cord sites activated by tragus stimulation, which may be a great oversight because, for example, the great auricular nerve is considered a superior branch of the cervical plexus (Ginsberg & Eicher, 2000). Indeed, neuronal tracing techniques, using cholera toxin B, of the primary afferent terminal projections from the tragus equivalent in the rat suggest that there is a major spinal afferent projection that merits further consideration (M Mohadi, J Deuchars and SA Deuchars, unpublished observations). These regions include the paratrigeminal nucleus and the dorsal horn of the spinal cord (Fig. 3). By stimulating a variety of sites within the ear, which are innervated to different extents by the great auricular nerve, auricular branch of the vagus or auriculotemporal nerve, we will elucidate the contributions of the different afferent nerves to the effects seen.

## Conclusion

In conclusion, non‐invasive neuromodulatory techniques have the potential to provide simple and inexpensive approaches to facilitating autonomic balance. Given that the pathways underlying the actions are not fully explored, further understanding is required to reveal how these may best positively influence autonomic circuits.

## Additional information

### Competing interests

None declared.

### Author contributions

This article was written by S.A.D. with editorial input from J.D. Experimental research was carried out and analysed by V.K.L., J.C., M.M., A.M. and L.P. with input from S.A.D. and J.D., and discussions during this research contributed to ideas within this symposium report. All authors approved the final version of the manuscript and agree to be accountable for all aspects of the work in ensuring that questions related to the accuracy or integrity of any part of the work are appropriately investigated and resolved. All persons designated as authors qualify for authorship, and all those who qualify for authorship are listed.

### Funding

Research conducted in these studies was supported by The British Heart Foundation (grant PG/08/120/26338 to S.A.D.), The Biotechnology and Biological Sciences Research Council (PhD studentship to V.K.L. and grant BB/E001831/1 to J.D.) and The Wellcome Trust (grant WT093072MA to S.A.D.).

### Present address

J. Clancy: School of Life Sciences, Thomson Building Room 347, University of Glasgow, Glasgow, G12 8QQ UK.
